# First-trimester urinary extracellular vesicles as predictors of preterm birth: an insight into immune programming

**DOI:** 10.3389/fcell.2023.1330049

**Published:** 2024-01-31

**Authors:** Jian-Pei Huang, Chia-Hsueh Lin, Chih-Wen Tseng, Ming-Hui Chien, Hung-Chang Lee, Kuender D. Yang

**Affiliations:** ^1^ Department of Obstetrics and Gynecology, Mackay Memorial Hospital, Taipei, Taiwan; ^2^ Department of Medicine, Mackay Medical College, New Taipei City, Taiwan; ^3^ MacKay Junior College of Medicine, Nursing and Management, New Taipei City, Taiwan; ^4^ Department of Medical Research, Mackay Memorial Hospital, Taipei, Taiwan; ^5^ Institute of Clinical Medicine, National Yang Ming Chiao Tung University, Taipei, Taiwan; ^6^ Mackay Children’s Hospital, Taipei, Taiwan

**Keywords:** preterm birth, first trimester, extracellular vesicles, biomarker, trained immunity, macrophage differentiation, T-cell regulatory differentiation

## Abstract

**Background:** The programming of innate and adaptive immunity plays a pivotal role in determining the course of pregnancy, leading to either normal term birth (TB) or preterm birth (PB) through the modulation of macrophage (M1/M2) differentiation. Extracellular vesicles (EVs) in maternal blood, harboring a repertoire of physiological and pathological messengers, are integral players in pregnancy outcomes. It is unknown whether urinary EVs (UEVs) could serve as a non-invasive mechanistic biomarker for predicting PB.

**Methods:** This study investigated first-trimester UEVs carrying M1 messengers with altered immune programming, aiming to discern their correlation to subsequent PB. A birth cohort comprising 501 pregnant women, with 40 women experiencing PB matched to 40 women experiencing TB on the same day, was examined. First-trimester UEVs were isolated for the quantification of immune mediators. Additionally, we evaluated the UEV modulation of “trained immunity” on macrophage and lymphocyte differentiations, including mRNA expression profiles, and chromatin activation modification at histone 3 lysine 4 trimethylation (H3K4me3).

**Results:** We found a significant elevation (*p* < 0.05) in the particles of UEVs bearing characteristic exosome markers (CD9/CD63/CD81/syntenin) during the first trimester of pregnancy compared to non-pregnant samples. Furthermore, UEVs from PB demonstrated significantly heightened levels of MCP-1 (*p* = 0.003), IL-6 (*p* = 0.041), IL-17A (*p* = 0.007), IP-10 (*p* = 0.036), TNFα (*p* = 0.004), IL-12 (*p* = 0.045), and IFNγ (*p* = 0.030) relative to those from TB, indicative of altered M1 and Th17 differentiation. Notably, MCP-1 (>174 pg/mL) exhibited a sensitivity of 71.9% and specificity of 64.6%, and MCP-1 (>174 pg/mL) and IFNγ (>8.7 pg/mL) provided a higher sensitivity (84.6%) of predicting PB and moderate specificity of 66.7%. Subsequent investigations showed that UEVs from TB exerted a significant suppression of M1 differentiation (iNOS expression) and Th17 differentiation (RORrT expression) compared to those of PB. Conversely, UEVs derived from PB induced a significantly higher expression of chromatin modification at H3K4me3 with higher production of IL-8 and TNFα cytokines (*p* < 0.001).

**Implications:** This pioneering study provides critical evidence for the early detection of altered M1 and Th17 responses within UEVs as a predictor of PB and early modulation of altered M1 and Th17 polarization associated with better T-cell regulatory differentiation as a potential prevention of subsequent PB.

## 1 Introduction

While pregnancy-related deaths and perinatal mortality rates have significantly decreased in developing nations, developed countries are experiencing an alarming increase in pregnancy-related fatalities and preterm births (PBs) (Fuchs et al., 2018; Ozimek and Kilpatrick, 2018; Rossen et al., 2020). Preterm birth, defined as delivery before 37 weeks of gestation, stands as a substantial public health concern. Although advancements have decreased preterm infant mortality rates, severe complications including cerebral injury, lung disease, and sepsis persist, imposing substantial burdens on families and society (Richter et al., 2019). Currently, there are limited strategies for predicting and preventing PB, with recent studies suggesting a modest reduction from 13.1% to 11.6% through low-dose aspirin intervention (Hoffman et al., 2020). PB can be triggered by maternal inflammation, placental insufficiency, or chromosomal anomalies (Wei et al., 2010; Kim et al., 2015; van Boeckel et al., 2018). The primary method for predicting PB involves monitoring the cervical length through transvaginal sonography every 2 weeks between 16 and 24 weeks of gestational age, although this approach lacks a clear mechanism for prevention (Ville and Rozenberg, 2018). Increased levels of the placental growth factor EG-VEGF have been demonstrated to play a role in parturition (Dunand et al., 2014) and shown as a predictor for PB in the second and third trimesters (Raia-Barjat et al., 2023). Other predictive factors for PB include cell-free fetal DNA (van Boeckel et al., 2018) and oxidative stress markers (Dunand et al., 2014) in blood samples taken close to the time of PB.

The orchestration of innate and adaptive immunity significantly influences pregnancy outcomes, directing either a normal term birth (TB) or PB by modulating macrophage (M1/M2) and T helper cell (Th1/Th2/Treg/Th17) differentiations. In a prior birth cohort study, we discovered associations between myeloperoxidase (MPO) and IL-8 levels in umbilical cord blood with PB, yet only IL-8 exhibited a correlation with infant cerebral palsy (Huang et al., 2004; Yang et al., 2004). However, this approach proves too late for effective PB prevention. Animal research has linked IL-1 to PB induction, demonstrating that the administration of an IL-1 receptor antagonist can prevent PB (Romero and Tartakovsky, 2004). Notably, at the onset of labor, type 1 macrophage (M1) differentiation is present in decidual tissue, suggesting its involvement in proinflammatory processes triggering labor compared to term pregnancies without labor (Xu et al., 2016). Pavlov et al. revealed higher levels of proinflammatory cytokines (IL-6 and TNFα) and lower levels of anti-inflammatory IL-10 in placental macrophages from term pregnancies, suggesting their involvement in parturition (Pavlov et al., 2008). IL-6-deficient mice exhibited resistance to endotoxin-induced PB, while IL-10 deficiency intensified inflammatory PB induction (Robertson et al., 2010; Lin et al., 2014). A Danish birth cohort study identified a correlation between mid-pregnancy plasma IFN-γ levels, indicative of type 1 T helper (Th1) response, and subsequent PB development (Curry et al., 2007). Collectively, these studies imply that altered M1 proinflammation and/or dominant Th1 response play a role in PB induction.

Extracellular vesicles (EVs), specifically exosomes ranging from 50 to 150 nm in size, serve as lipid-bilayer membrane carriers facilitating cellular communication and participating in both physiological and pathological functions, including fetal–maternal interactions (Guo et al., 2023). These minuscule vesicles are released into the bloodstream and urine, playing significant roles in fetal–maternal disorders such as gestational hypertension, gestational diabetes mellitus, preterm birth, and fetal growth restriction (Ghafourian et al., 2022). EVs in maternal blood, harboring a repertoire of physiological and pathological messengers, are integral players in pregnancy outcomes. However, the presence of mechanistic immune mediators within maternal urinary EVs (UEVs) involved in PB remains unclear. McElrath et al. demonstrated that maternal plasma-derived EVs hold a proteomic signature for early preeclampsia prediction before 35 weeks of gestational age (McElrath et al., 2020). Additionally, a distinct proteomic profile of plasma EVs derived from the late first trimester proved effective in predicting PB compared to those derived from term births (McElrath et al., 2019). However, the mechanisms underlying these unique protein profiles associated with PB and preeclampsia remain unknown, and mass spectrometry-identified proteins often prove challenging to validate through functional or immune assays.

Given the shared developmental origins and communication between the uro-system and genito-system, it is noteworthy that most preterm births are linked with high-risk pregnancies or intrauterine growth retardation, conditions associated with fetal–maternal disorders correlated to exosomal biomarkers in maternal circulation (Yang et al., 2019; Czernek and Düchler, 2020; Libretti and Aeddula, 2020). In our pursuit of mechanistic biomarkers utilizing UEVs from first-trimester urine as a liquid biopsy, recent investigations revealed that UEVs derived from young individuals exhibited lower proinflammatory cytokines but higher defense mediators compared to those from older adults (Yeh et al., 2021). Studies in animal models suggest that EVs derived from macrophages play a role in mediating placental inflammation (Holder et al., 2016), and the “trained immunity” of innate immune cells at the fetal–maternal interface possessed the ability to “remember” for subsequent successful pregnancies (Feyaerts et al., 2021). Furthermore, research indicates that the trained immunity of M1 proinflammation induced by *Bacillus Calmette–Guérin* (BCG) could restrict fetal growth in animal models (Dang et al., 2022) and decorin-induced polarization of decidual type 1 macrophage (M1) differentiation via mitochondrial dysfunction, resulting in recurrent pregnancy loss (Wang et al., 2022). In this study, we collected first-trimester urine samples from pregnant women and assessed the levels of various immune mediators in UEVs. We subsequently compared these levels between women who later experienced PB and those who delivered at term. Additionally, we investigated whether first-trimester UEVs from women with PB and TB exhibited differential effects on suppressing the trained immunity with M1 proinflammation and/or T-cell differentiation, aiming to identify the mechanistic biomarkers of UEVs for subsequent PB and potentially propose preventive regimens.

## 2 Materials and methods

### 2.1 Study design and subjects

This is a birth cohort study. Pregnant women were enrolled in the study after obtaining informed consent. The study was approved by the Institutional Review Board of Mackay Memorial Hospital, Taiwan (Approval No. 21MMHIS061e). We estimated that a birth cohort of 400 pregnant participants giving birth to 40 preterm babies would provide the study a power of over 80% under an effect size of 0.4 (e.g., 40% difference of certain cytokine levels) and alpha value of 0.05. We completed the recruitment of 501 pregnant women and identified 40 preterm births. Each first-trimester (gestational age between 12 and 14 weeks) urine sample of the 40 women with PB was individually paired with the first-trimester urine sample of the other 40 women with TB in the same day and subjected to isolation of UEVs for the measurement of the differential displays of immune mediators and immune functions between the first-trimester UEVs from PB and TB.

### 2.2 Isolation of UEVs and tracking particles and size by nanoparticle tracking analysis

We isolated UEVs using a size exclusion filtration system, as previously described (Yeh et al., 2021). In brief, the urine samples (30 mL) were collected in the first trimester and stored at −20°C until further study. To isolate UEVs, the frozen urine samples were rapidly thawed at 37°C and centrifuged at 3,000 *g* for 10 min at 4°C to remove cell debris. The cell-free urine was passed through a 1.0-µm polyethersulfone (PES) filter and another 0.22-µm filter to remove large EVs, followed by final separation using a 0.03-µm regenerated cellulose (RC) filter to obtain the UEVs. The UEVs were harvested by washing with PBS three times and adjusted to a condensation of 200-fold (30 mL input of urine and 150 µL output of UEVs). The output UEVs were aliquoted to 50 µL (three vials) and stored at −70°C until further study. To measure the particles and size of UEVs, we used a NanoSight nanoparticle tracking analyzer (NTA) (Inning, Germany). The measurement was optimized in a dilution factor of 1:100, 1:500, 1:1,000, or 1:5,000 to make the UEVs detectable between 10^7^ and 10^8^ vesicles/ml in triplicate (Shiue et al., 2019; Tsai et al., 2021; Yeh et al., 2021). The data for the initial 3 min of tracing were not recorded because of unstable flow tracing. The data on the vesicle size vs. concentration curve were acquired between 3 and 6 min of tracing.

### 2.3 Characterization of UEVs by Western blot analyses

To characterize the UEVs possessing exosomal markers, we subjected the UEVs (5 × 10^9^ EVs/20 μL; ∼ 2 µg) obtained from pregnant and non-pregnant women to 10% polyacrylamide gel electrophoresis. Due to the lack of a definitive housekeeping marker for UEVs, we used an equal amount of UEVs (5 × 10^9^ EVs/20 μL) for Western blot analysis despite including syntenin as a presumptive housekeeping marker of UEVs. The protein electrophoresis gel was transferred to a PVDF membrane, washed with Tris buffer, and blocked with 5% nonfat dry milk in the Tris buffer with 0.1% Tween 20 before being subjected to primary antibodies directed against CD9, CD81, CD63, or syntenin overnight at 4°C (Shiue et al., 2019; Tsai et al., 2021; Yeh et al., 2021). After washing three times with Tris buffer, the biotin-conjugated secondary antibody at a 1:5,000 dilution was incubated for 30 min at room temperature. Finally, the blots were washed and exposed to streptavidin and analyzed using an electrochemiluminescence (ECL) kit for visualizing the fluorescent protein density.

### 2.4 Immune mediators of UEVs measured by multiplex bead arrays

The cytokines and chemokines in the UEVs were measured by MILLIPLEX^®^ bead arrays. In brief, UEV samples (25 ul), which presented the total particles of UEVs between 9.25 × 10^9^ and 10.25 × 10^9^ cells, were lysed by RIPA buffer at a 1:2 ratio, followed by the addition of 25-μL fluorescent beads conjugated with specific antibodies directed against different cytokines, IFN-α2, IP-10, TNFα, IL-6, IL-8, MCP-1, IL-12p40, IFNγ, IL-4, IL-15, IL-17A, IL-10, and IL-13. The plate was sealed and wrapped with foil for agitation on a plate shaker overnight at 4°C. The plate was washed three times and incubated with 25 μL of the secondary antibody conjugated with phycoerythrin (PE) for another 30 min at RT. The plate underwent a final series of washes before 150 μL of sheath fluid was added for the measurement on the Bio-Plex 200 system (Bio-Rad Laboratories, Hercules, CA). Quality control values for each standard marker were consistently shown within the range indicated by the manufacturer (Yeh et al., 2021).

### 2.5 Modulation of trained immunity by the UEVs derived from women with PB and TB

To assess the mechanism in altered immune mediators and functions of trained immunity by UEVs derived from women with PB and TB, we investigated the effects of UEVs on the trained immunity of M1/M2 and Th1/Th2/Treg/Th17 differentiation of cell surface markers of differentiation, differential mRNA expression, and chromatin modification. The BCG-trained immunity was modified from a previous report, in which both innate and adaptive immunity were studied in the BCG-trained immunity (Mills, 2012; Kleinnijenhuis et al., 2014; Li et al., 2015). Peripheral blood mononuclear cells (PBMCs) obtained from non-pregnant women (aged from 25 to 35 years) were isolated by density gradient centrifugation, resuspended to 2 × l0^6^ cells/mL in RPMI 1640 medium, and subjected to the incubation of BCG at 5 ug (5 × 10^4^ CFU) for 1 day, followed by training for 5 days after washing out the incubation and the final 1 day of lipopolysaccharide (100 ng/mL) stimulation. The culture supernatants were harvested for the measurement of glucose consumption by using GM700SB (Bionime Co., Ontario, CA) and the measurement of immune mediators by MILLIPLEX^®^ bead assay, as described above, and the PBMCs were divided into two portions for total RNA and total protein extractions, which were subjected to measurements of the mRNA expression of M1/M2 differentiation and transcription factor mRNA expression of Th1/Th2/Treg/Th17 differentiation by qRT–PCR, respectively, and chromatin activation modification at histone 3 lysine 4 trimethylation (H3K4me3) by Western blot analysis.

### 2.6 qRT–PCR measurement of the differential mRNA expression of M1/M2 and Th1/Th2/Treg/Th17 differentiation

PBMCs (1 × 10 ^6^ cells/mL) under different conditions were harvested and subjected to total RNA extraction using TRI Reagent solution (Sigma, St. Louis, MO, United States). The total RNA samples were isolated by isopropanol precipitation and resuspended in 20 ul of DEPC-treated H_2_O. These RNA samples were subjected to real-time quantitative RT–PCR of M1/M2 and Th1/Th2/Treg polarization. The reverse transcription (RT) was performed using a master assay kit containing both random and oligo-dT primers with 50 ng input RNA and incubated at 65°C for 2 min before adding 0.5 μL of MMLV reverse transcriptase for 1 h at 37°C. After the RT step, the PCR was performed using 5 μL of cDNA, the 10 μL 2X SYBR Green Master Mix, 1.0 μL (10 nM) forward and reverse primers, and DEPC water to obtain a total of 20 μL. The thermal cycling for qPCR was performed as follows: 95°C for 1 min and 40 cycles of denaturing at 95°C for 5 s and 60°C for 30 s. The M1/M2 polarization was assessed by the mRNA expression of iNOS and arginase 1 (ARG1), which reciprocally represent M1 and M2 differentiation (Bermick et al., 2017). The Th1/Th2/Treg/Th17 differentiation was assessed by a qRT–PCR analysis of transcription factors T-bet, Gata-3, RORrT, and FoxP3 mRNA expression. The forward and reverse primers for qRT–PCR of M1/M2 and Th1/Th2/Treg/Th17 differentiation are given in Table 1.

**TABLE 1:** Primers for measuring mRNA expression of M1/M2 and Th1/Th2/Treg/Th17 differentiation.

Markers	Forward (5'-3')	Reverse (5'-3')
M1/M2		
iNOS	GCT​GTA​TTT​CCT​TAC​GAG​GCG​AAG​AA	CTT​GTT​AGG​AGG​TCA​AGT​AAA​GGG​C
ARG1	TCC​AAG​GTC​TGT​GGG​AAA​AG	ATT​GCC​AAA​CTG​TGG​TCT​CC
GAPDH	GAG​TCA​ACG​GAT​TTG​GTC​GT	TTG​ATT​TTG​GAG​GGA​TCT​CG
Th1/Th2/Treg		
T-bet	AAC​ACA​GGA​GCG​CAC​TGG​AT	TCT​GGC​TCT​CCG​TCG​TTC​A
GATA3	GTG​CTT​TTT​AAC​ATC​GAC​GGT​C	AGG​GGC​TGA​GAT​TCC​AGG​G
FOXP3	CAA​GTT​CCA​CAA​CAT​GCG​AC	ATT​GAG​TGT​CCG​CTG​CTT​CT
RORrT	TTT​TCC​GAG​GAT​GAG​ATT​GC	CTT​TCC​ACA​TGC​TGG​CTA​CA
RPL13A	CGA​GGT​TGG​CTG​GAA​GTA​CC	CTT​CTC​GGC​CTG​TTT​CCG​TAG

In the PCR, SYBR Green was used as an intercalating DNA dye for measuring the fluorescence threshold, which was manually set across all samples in each experiment, starting with the exponential phase of the fluorescent signal increase at 10 times. The fractional number of RT–PCR cycles presenting the fluorescent tracing over the tracing of the negative control reaction was defined as the cycle threshold (Ct). The quantity of the iNOS and ARG1 mRNA expression presenting M1 and M2, respectively, was calculated based on each Ct value of the reactions, which was normalized to the internal control mRNA expression, as shown by the equation at 2 ^{[Ct 1 (target 1) – Ct 1 (GAPDH)] – [Ct2 (target 2) – Ct2 (GAPDH)]}^. Ctl (target 1) and Ct2 (target 2) represent the Ct values for the target gene expression with and without UEV treatment. Similarly, the quantity of the T-bet, Gata-3, FoxP3, and RORrT mRNA expression presenting Th1, Th2, Treg, and Th17, respectively, was calculated based on each Ct value of the reactions, which was normalized to the internal control RPL13A mRNA expression, as shown by the equation at 2 ^{[Ct 1 (target 1) – Ct 1 (RPL13A)] – [Ct2 (target 2) – Ct2 (RPL13A)]}^. Ct1 (target 1) and Ct2 (target 2) represent the Ct values for the target gene expression with and without UEV treatment.

### 2.7 Histone modification on the H3K4me3 expression of the trained immunity by UEVs from women with PB and TB

The total proteins harvested from PBMCs (2 × 10^6^ cells/mL) under different conditions were subjected to protein extraction using RIPA buffer. After measuring the protein amounts from cells under different conditions, 20 µg of proteins were loaded to 10% gel electrophoresis. After the protein gel electrophoresis, the protein gels were transferred to a PVDF membrane, washed with Tris buffer, and blocked with 5% nonfat dry milk in Tris buffer with 0.1% Tween 20 before adding the specific antibodies directed against H3K4me3 (Millipore 04-745) compared to the internal control antibody directed against GAPDH at 1:1,000 dilution (100 ng/mL) for 24 h at 4°C. After washing three times in Tris buffer, the biotin-conjugated secondary antibody at a 1:2,000 dilution was incubated for 30 min at room temperature. Finally, the blots were washed and exposed to streptavidin and analyzed using an ECL kit for visualizing the fluorescent density of protein gels.

### 2.8 Data acquisition and statistics

The demographic data of pregnant women were obtained after informed consent. We normalized the levels of immune mediators in UEVs with urine creatinine levels (mg creatinine/mL), which are measured by the reaction with alkaline picrate solution (R&D Systems, MN, United States) at 1:20 dilution for 30 min and read by OD_490nm_ because the concentration of immune mediators in UEVs could be affected by urinary dilution or condensation. We presented data for the qRT–PCR detection of the M1/M2 differential mRNA expression of iNOS and ARG1 after normalization with an internal control of GAPDH expression. We presented transcription factor (T-bet/Gata-3/FoxP3/RORrT mRNA) expression for the qRT–PCR detection of Th1/Th2/Treg/Th17 differentiation after normalization with an internal control of RPL13A expression because of its much better reference for the qRT–PCR of gene expression in human T lymphocytes (Li et al., 2015). We expressed the results with parametric measurements as the means ± SE and analyzed them using Student's t-test for comparing the two groups with normal distribution and using one-way ANOVA, followed by *post hoc* tests for comparison among groups. We expressed the results with non-parametric measurements as ratios and analyzed them using the chi-squared test, expressed the results with parametric data without normal distribution as arbitrary units ± SE, and analyzed them using the Mann–Whitney U test. We assessed the sensitivity and specificity for the prediction of PB by immune mediators in UEVs after cutting off the threshold at one standard deviation level, and a *p*-value of 0.05 or less was considered statistically significant. We performed all statistical analyses using the Statistical Package for Social Sciences (SPSS Inc., Chicago) version 17.0 for Windows.

## 3 Results

### 3.1 Demographic data of pregnant women with PB and TB

In this birth cohort, we recruited 501 pregnant women between September 2019 and October 2022 to participate in the study, collecting first-trimester urine samples and following up to the subsequent TB or PB. Among them, 40 women experienced PB. They were individually matched with 40 women with TBs for comparative analysis. While gestational age at birth significantly differed between the PB and TB groups (*p* < 0.0001), other demographic factors including age, weight, primiparity rate, gestational diabetes rate, cervix length between 20 and 28 weeks, and mild echography abnormalities showed no significant differences (Table 2).

**TABLE 2:** Demographic data of pregnant women with PB and TB.

Maternal variables	PB (n = 40)	TB (n = 40)	*p-*values
Age (years)	31.5 ± 0.7 (21–39)	33.5 ± 0.7 (24–44)	>0.05
Weight (kg)	58.6 ± 2.4 (40–104)	60.7 ± 2.1 (42–115)	>0.05
Gestational age at delivery (weeks)	33.7 ± 0.8 (16–36.6)	39.1 ± 0.2 (37.4–41)	<0.0001*
Primiparity	24/40	20/40	>0.05
Gestational diabetes mellitus	4/40	5/40	>0.05
Cervix length (<3.0 cm)	3/40	0/40	>0.05
Ultrasound abnormalities	#8/40	3/40	>0.05

Data on age, weight, and gestational age were subjected to analysis using Student’s t-test for parametric data, while rates of primiparity, gestational diabetes, cervix length, and mild ultrasound abnormalities underwent analysis using the chi-squared test for non-parametric data. Significance is denoted by *, and # indicates the count of mild ultrasound abnormalities at eight in the PB group, namely, short limbs, mild–low amniotic fluid index, oligohydramnios, placenta previa partialis, increased uterine artery pulsatility index, and myoma, three in the TB group, namely, left-ventricular echogenic foci, mild hydronephrosis, and mild enlarged uterus and adnexa.

### 3.2 Number, size, and characteristics of UEVs among urine samples from women with and without pregnancy

**FIGURE 1:**
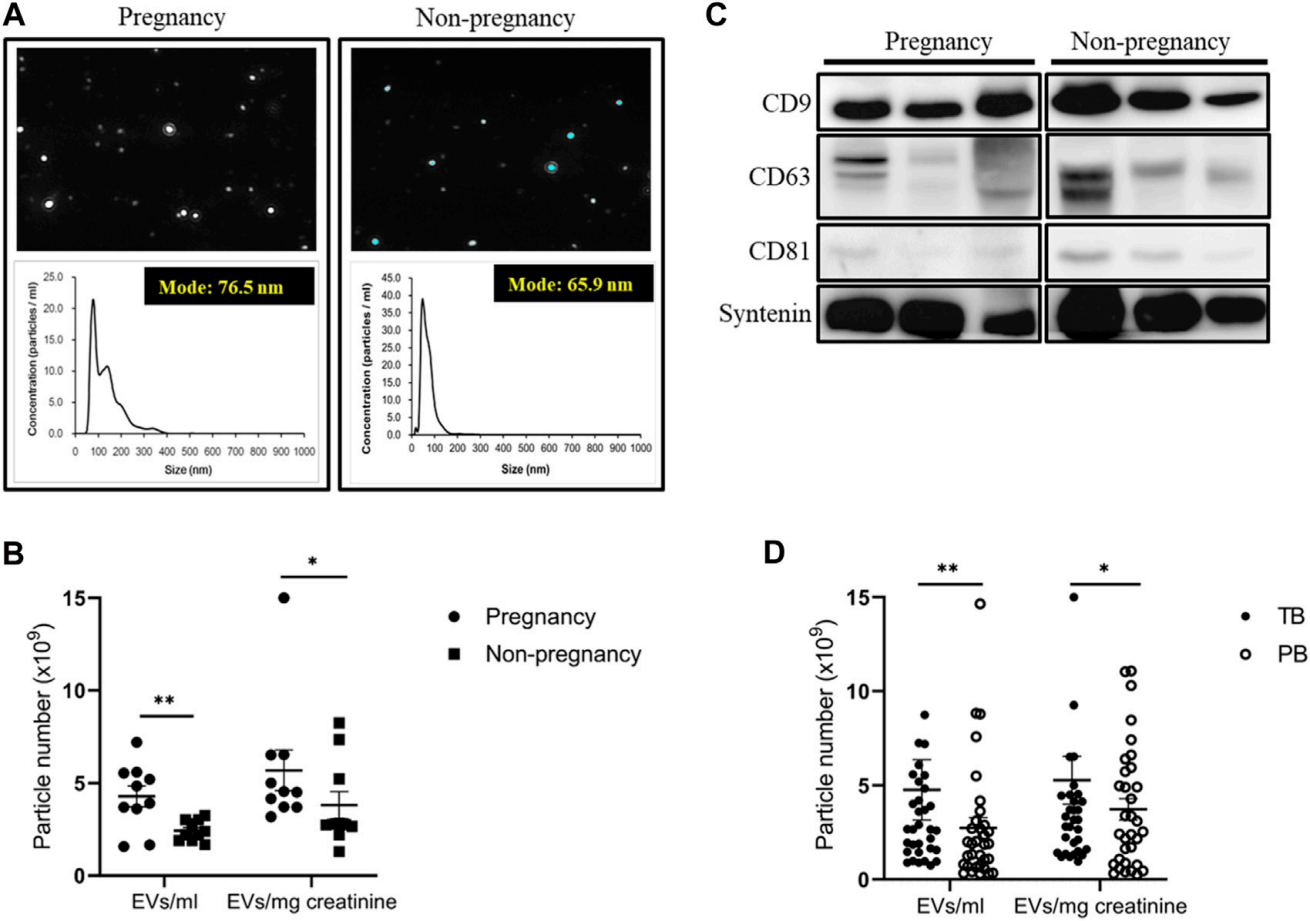
Number and characteristics of UEVs obtained from women with and without pregnancy. **(A)** Higher number of UEVs from women with pregnancy, associated with a larger size with an average mode of 76.5 nm compared to those (average 65.9 nm) from women without pregnancy in the NTA (n = 10). **(B)** The mean vesicle count was significantly higher (*p* < 0.05) in UEVs from pregnant women, regardless of whether correction for urinary dilution or condensation was carried out based on urine creatinine levels (mg/mL). **(C)** The characteristic expression of exosome markers (CD9/CD63/CD81/syntenin) was comparable between first-trimester UEVs derived from women with and without pregnancy. In this Western blot analyses of exosomal markers in UEVs, the samples were loaded with a standardized quantity of particles (5 × 10^9^ EVs/20 μL) for UEV characterization, and syntenin was used as a putative housekeeping marker for UEVs. **(D)** The mean vesicles were significantly higher (*p* < 0.05) in UEVs from women with full-term birth (TB; n = 40) than those with preterm birth (PB; n = 40), irrespective of correction for urinary dilution or condensation by urine creatinine levels (mg/mL). * indicates *p* < 0.05, and ** indicates *p* < 0.01.

Using an NTA, we observed a substantial increase in UEVs in pregnant women (N = 10, mean age 32.0 years) compared to non-pregnant women (N = 10, mean age 30.5 years) when quantified directly in vesicles per milliliter or after adjusting for urine concentration by normalizing to urine creatinine levels in vesicles per milligram of creatinine (mg/mL). The UEVs exhibited a diverse range of sizes, with averaged modes at 65.9 nm and 75.9 nm for non-pregnant and pregnant women, respectively (Figure 1A). The mean vesicle counts were 5.7 × 10^9^ ± 1.1 × 10^9^ and 3.8 × 10^9^ ± 7.4 × 10^8^ (EVs/mg creatinine/mL) in the UEVs derived from women with and without pregnancy, respectively, which are significantly different (*p* < 0.05) between the two groups (Figure 1B). The expression levels of exosomal characteristic markers (CD9, CD63, CD81, and syntenin) were comparable between pregnant and non-pregnant women, as demonstrated by the comparison of three representative pairs (Figure 1C). Interestingly, although the mode of sizes was not significantly different between UEVs from PB and TB women, the vesicle counts of UEVs from PB (3.7 × 10^9^ ± 5.6 × 10^8^ EVs/mg creatinine/mL) were significantly lower (*p* < 0.05) than those from TB (5.3 × 10^9^ ± 1.3 × 10^9^ EVs/mg creatinine/mL) (Figure 1D).

### 3.3 Immune mediators of first-trimester UEVs between women with PB and TB

Employing a multiplex bead assay, we assessed the immune mediators within UEVs. Our findings revealed that UEVs obtained during the first trimester from women with PB exhibited significantly heightened levels of M1 cytokines TNFα (*p* = 0.004), MCP-1 (*p* = 0.003), and IP-10 (*p* = 0.036), Th1 cytokines IL-12 (*p* = 0.045) and IFNγ (*p* = 0.030), and Th17 cytokines IL-6 (*p* = 0.041) and IL-17A (*p* = 0.001), compared to those from women with TB (Table 3). These elevations were indicative of M1 (TNFα, MCP-1, and IP-10), Th1 (IL-12 and IFNγ), and Th17 (IL-6 and IL-17A) polarization. Upon setting the threshold at one standard deviation point, we found that MCP-1 (>174 pg/mL) exhibited a sensitivity of 71.9% and specificity of 64.6%, while IFNγ (>8.7 pg/mL) displayed a moderate sensitivity (57.6%) but higher specificity (85.7%) in predicting PB. Additionally, the combination of MCP-1 (>174 pg/mL) and IFNγ (>8.7 pg/mL) provided a higher sensitivity (84.6%) in predicting PB, with a moderate specificity of 66.7% (Table 4).

**TABLE 3:** Immune mediators of UEVs predicting PB compared to TB.

Cytokine	UEVs of PB	UEVs of TB	T-test
IFN-α2	247 ± 56	166 ± 42	0.1252
IFN-y	69 ± 14	36 ± 9	0.0298
IL-10	676 ± 127	444 ± 122	0.0950
IL-12p40	10 ± 3	4 ± 1	0.0448
IL-13	10 ± 3	9 ± 2	0.4054
IL-15	62 ± 10	44 ± 11	0.1058
IL-17A	7 ± 1	3 ± 0	0.0007
IL-la	8 ± 4	8 ± 4	0.4541
IL-4	230 ± 64	675 ± 437	0.1596
IL-6	33 ± 16	4 ± 1	0.0405
IL-8	147 ± 53	74 ± 66	0.1951
IP-10	134 ± 66	12 ± 4	0.0356
TNFa	4 ± 1	2 ± 0	0.0044
MCP-1	1,169 ± 325	191 ± 72	0.0027

Notes: Cytokine concentrations are presented in pg/mL following adjustment of urine creatinine levels (mg/mL).

**TABLE 4:** Immune mediators of UEVs predicting PB compared to TB and the sensitivity and specificity of predicting PB.

Cytokines	PB	TB	Prediction
MCP-1 > 174 pg/mL	n = 40	n = 40	Sensitivity and specificity
Positive	23	9	71.9% (23/32)
Negative	17	31	64.6% (31/48)
IFN-y > 8.7 pg/mL	n = 40	n = 40	Sensitivity and specificity
Positive	38	28	57.6% (38/66)
Negative	2	12	85.7% (12/14)
MCP-1 and IFN-y	n = 40	n = 40	Sensitivity and specificity
Positive	22	4	84.6% (22/26)
Negative	18	36	66.7% (36/54)

Forty pairs of first-trimester UEVs obtained from women with PB or TB were assessed using a multiplex bead assay. A) Cytokine concentrations are presented in pg/mL following adjustment for urine creatinine levels (mg/mL). The disparity in cytokine levels between PB and TB was assessed using Student's t-test. B) Sensitivity and specificity for predicting PB were determined based on cut-off points set at one standard deviation from the mean of the two comparative groups. Specifically, cut-off points for MCP-1 and IFNγ were established at 174 pg/mL and 8.7 pg/mL, respectively, for predicting PB.

### 3.4 Effects of UEV modulation on trained immunity with M1/M2 differentiation

**FIGURE 2:**
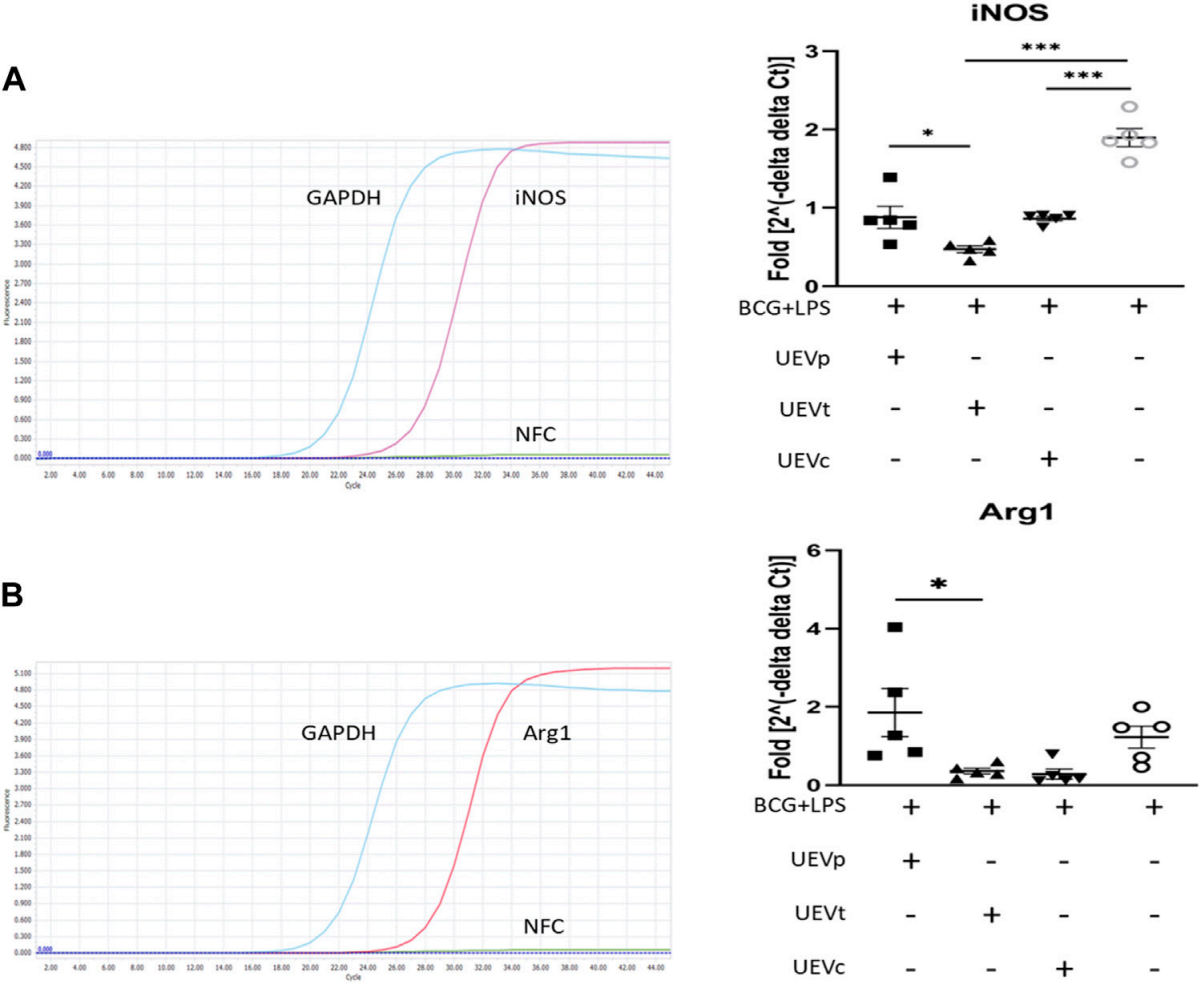
UEV modulation of M1/M2 differentiation on iNOS and Arg1 mRNA expression. **(A)** Representative qRT–PCR results with specific iNOS primers and internal control GAPDH primers (left column). Among five experiments, the induction of trained immunity by BCG and LPS resulted in the highest iNOS expression. UEVs derived from women with TB (UEVt) or those from non-pregnant controls (UEVc), but not UEVs from PB (UEVp), significantly downregulated iNOS expression (right column). **(B)** Representative qRT–PCR results with specific Arg1 primers and internal control GAPDH primers (left column). Among five experiments, induction of trained immunity by BCG for 6 days, followed by LPS stimulation for 1 day, led to the highest Arg1 expression. UEVs derived from TB (UEVt) or those from non-pregnant controls (UEVc), but not UEVp, significantly suppressed Arg1 expression (right column). * represents a *p*-value < 0.05, and *** indicates a *p*-value < 0.001, as tested using an ANOVA model using *post hoc* tests.

Given the established link between the trained immunity involving memory macrophages or natural killer (NK) cells and pregnancy outcomes (Feyaerts et al., 2021; Dang et al., 2022; Wang et al., 2022), we explored whether UEVs obtained from PB and TB women exerted distinct effects on trained immunity, particularly in the context of M1/M2 differentiation. By implementing a protocol involving the trained immunity induction with BCG for 6 days, followed by LPS stimulation for 1 day, we observed a notable enhancement of M1 differentiation, evidenced by elevated iNOS mRNA expression. Furthermore, our findings indicated that UEVs sourced from TB (UEVt) or those from non-pregnant control (UEVc) women exhibited a more pronounced suppressive effect on M1 differentiation (*p* < 0.001 (Figure 2A). UEVt appeared to suppress iNOS expression more prominently than those derived from PB (UEVp) (*p* = 0.026; Figure 2A). This observation aligns with significantly lower levels of MCP-1, TNFα, and IL-6 in UEVs from TB compared to those from PB (Table 3). Additionally, UEVs obtained from TB (UEVt) significantly attenuated Arg1 expression to a greater extent than those from UEVp (*p* = 0.043; Figure 2B).

### 3.5 UEV modulation of Th1/Th2/Treg/Th17 differentiation

**FIGURE 3:**
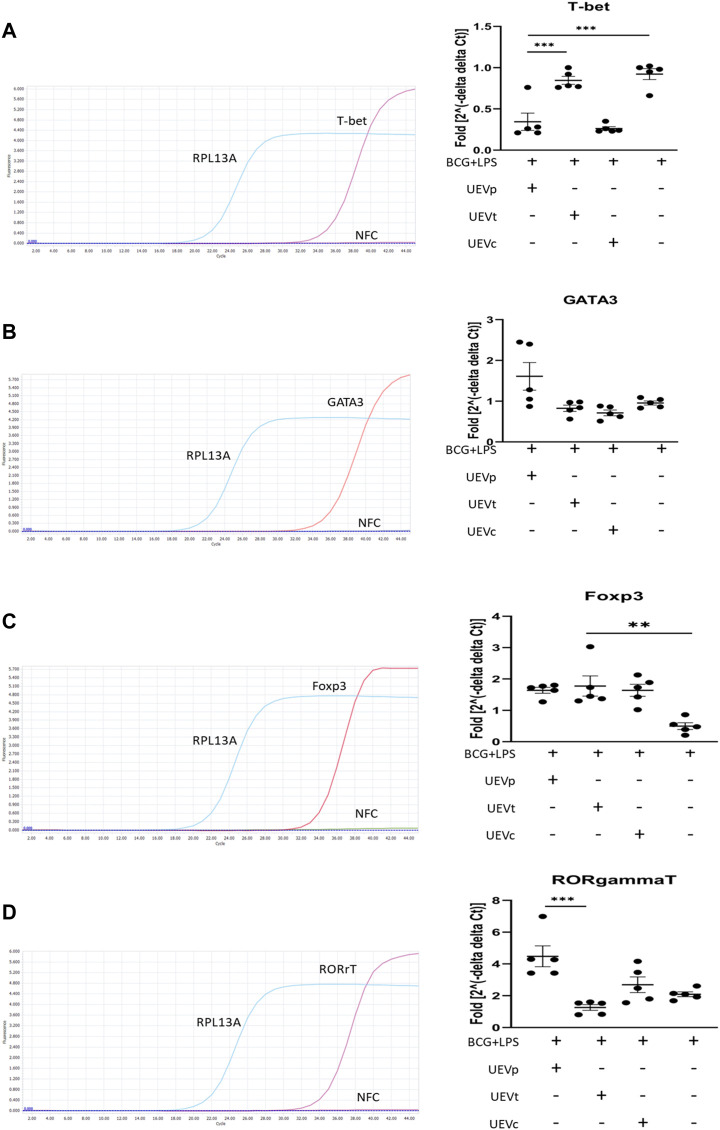
UEV modulation of Th1/Th2/Treg/Th17 differentiation on T-bet, Gata-3, FoxP3, and RORrT transcription factor expression. **(A)** Representative qRT–PCR results with specific T-bet primers and internal control RPL13A primers (left column). Across five experiments, induction of trained immunity with BCG for 6 days, followed by LPS stimulation for 1 day, did not result in a higher T-bet expression, but UEVs derived from TB (UEVt) appeared to induce higher T-bet expression than those from non-pregnant control (UEVc) or UEVs from PB (UEVp) (right column). **(B)** Representative qRT–PCR results with specific Gata-3 primers and internal control RPL13A primers (left column). In five experiments, trained immunity with BCG and LPS did not lead to enhanced Gata-3 expression, and UEVs derived from PB (UEVp) did increase the Gata-3 expression, although not significantly (right column). **(C)** Representative qRT–PCR results with specific FoxP3 primers and internal control RPL13A primers (left column). In five experiments, trained immunity with BCG and LPS had the lowest FoxP3 expression. However, UEVp, UEVt, or UEVc derived from non-pregnant controls induced higher FoxP3 expression (right column). **(D)** Representative qRT–PCR results with specific RORrT primers and internal control RPL13A primers (left column). Across five experiments, trained immunity with BCG and LPS induced a 2-fold increase of RORrT expression, and UEVs derived from PB (UEVp) induced a significantly higher RORrT expression than the UEVs from TB (UEVt). * represents a *p*-value < 0.05, and *** indicates a *p*-value < 0.001, as tested using an ANOVA model using *post hoc* tests.

We also explored the impact of UEVs on trained immunity, focusing on Th1/Th2/Treg/Th17 differentiation. Following a 6-day induction of trained immunity with BCG and 1 day of LPS stimulation, we observed significant Th1 differentiation, evident from the elevated T-bet transcription factor expression normalized to the internal control RPL13A mRNA expression. Notably, UEVs obtained from PB (UEVp) demonstrated a more potent suppressive effect on Th1 differentiation (T-bet expression) than those from TB (Figure 3A). While trained immunity did not lead to heightened Gata-3 expression, UEVp enhanced Gata-3 expression, although not significantly different from the levels induced by UEVt or UEVc (Figure 3B). Both UEVs obtained from PB and TB significantly promoted Treg differentiation, as indicated by higher FoxP3 expression (*p* < 0.003; Figure 3C). In contrast, UEVp, but not UEVt or UEVc, significantly enhanced Th17 expression (RORrT expression) (*p* < 0.001; Figure 3D), highlighting that UEVs derived from PB could skew toward higher Th17 differentiation, corroborating with the elevated levels of IL-6 and IL-17A observed in UEVs from PB (Table 3).

### 3.6 UEV inhibition of trained immunity with glycolysis

**FIGURE 4:**
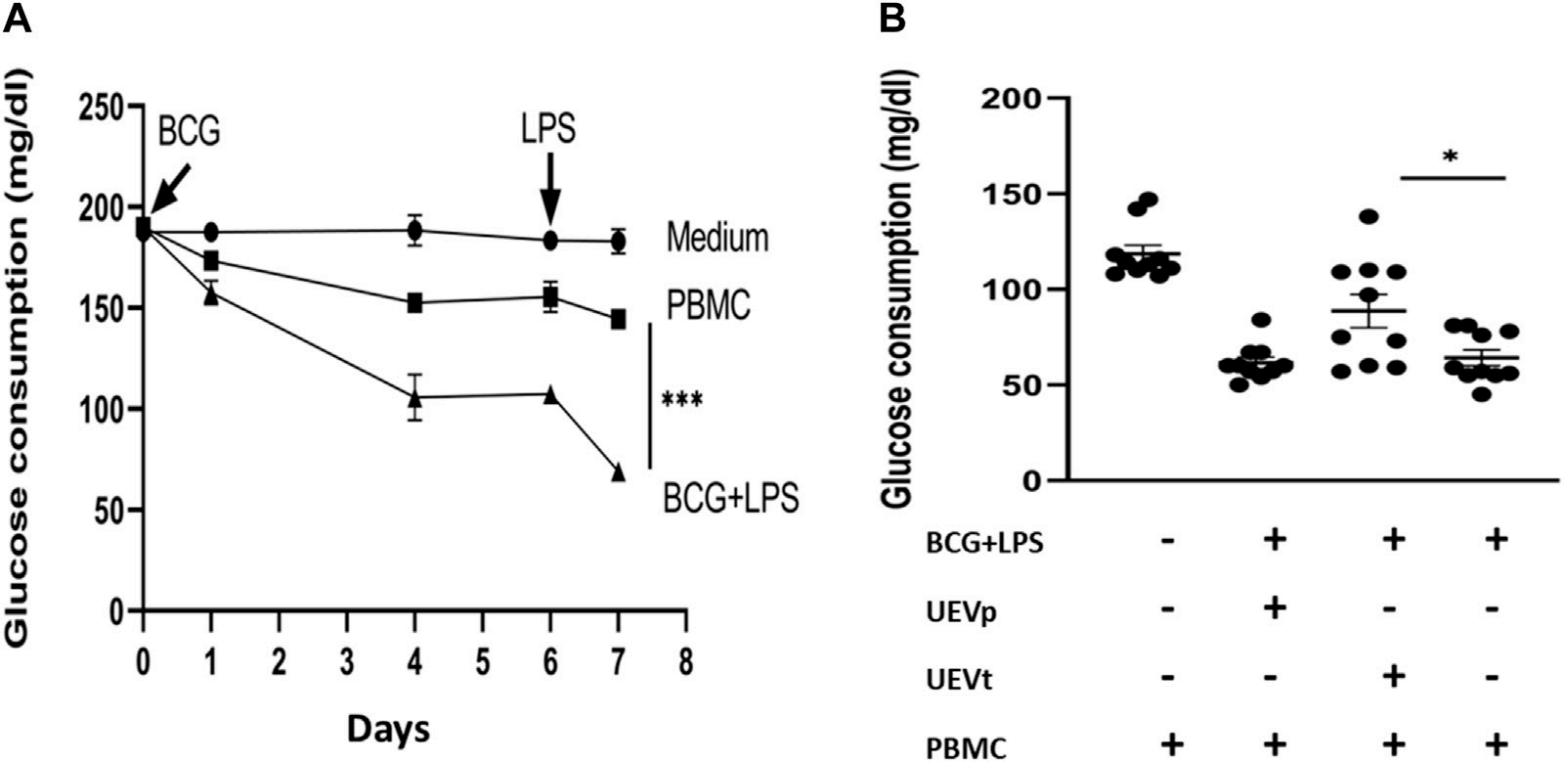
UEVt reversed the trained immunity-associated glucose consumption. Using the trained immunity by BCG for 6 days, followed by the stimulation of LPS for 1 day, we found that the trained immunity by BCG and LPS induced the highest glucose consumption (n = 10, *p* < 0.001) **(A)**. While comparing the effects between UEVp and UEVt on glucose consumption, we found that UEVt but not UEVp significantly (*p* = 0.020, n = 10) inhibited the glucose consumption by the trained immunity **(B)**. * represents a *p*-value < 0.05, and *** indicates a *p*-value < 0.001, as tested using an ANOVA model using *post hoc* tests.

Subsequent experiments were conducted to investigate the potential link between glycolysis and the UEV-mediated modulation of trained immunity, induced by BCG for 6 days followed by an additional day of LPS exposure. The fresh RPMI 1640 media contained glucose concentrations between 181 and 186 mg/dL (mean = 184 mg/dL). The PBMCs without trained conditions revealed a relatively lower consumption of glucose, and the trained immunity induced by BCG and LPS induced a significant increase in glucose consumption (*p* < 0.001) (Figure 4A). UEVt, but not UEVp, reversed the heightened glucose consumption associated with the trained immunity induced by BCG and LPS (*p* = 0.020) (Figure 4B).

### 3.7 Different effects of UEVp and UEVt on activating chromatin modifications

**FIGURE 5:**
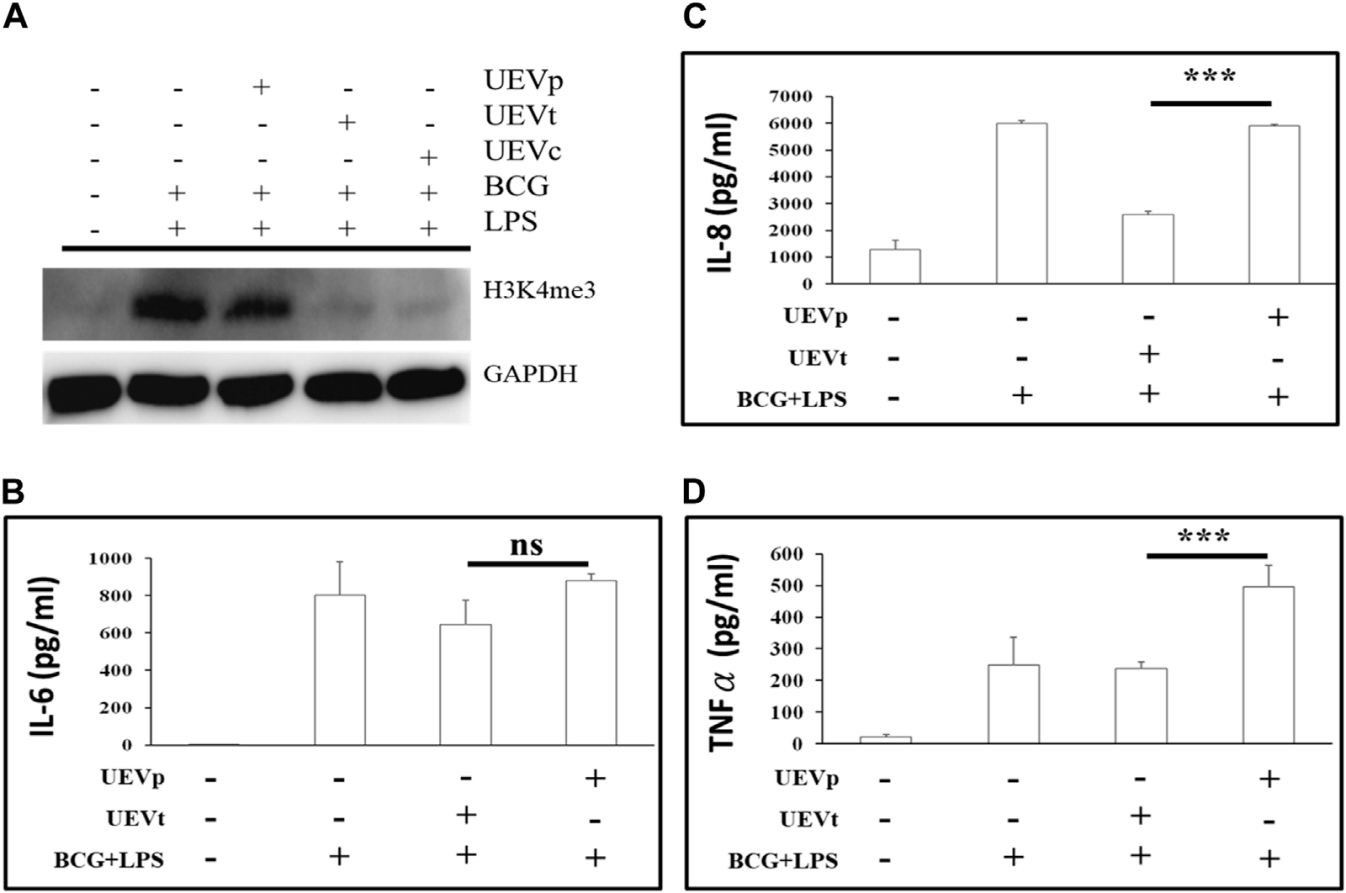
UEV modulation of chromatin activation modification at H3K4me3 expression and cytokine production. PBMCs were subjected to the trained immunity with and without UEV modulation. After the trained immunity, the culture supernatants were collected for the measurement of cytokine production, and PBMCs were harvested for Western blot analyses of H3K4me3 expression. **(A)** A representative Western blot of the H3K4me3 expression, derived from four reproducible experiments (see the blots in Supplementary File), was prominently induced after the trained immunity by BCG and LPS (A: lanes 1 and 2). UEVs obtained from TB (UEVt, lane 4) inhibited H3K4me3 accumulation better than those from PB (UEVp, lane 3). The trained immunity by BCG and LPS was accompanied with higher IL-6 **(B)**, IL-8 **(C)**, and TNFα **(D)** production in five experiments. The UEVp, which did not suppress the H3K4me3 expression, promoted the higher production of proinflammatory cytokines IL-8 **(C)** and TNFα **(D)** than those from TB (UEVt). *** indicates a *p*-value < 0.001, as tested using the Mann–Whitney U test.

It is established that trained immunity induced by BCG and LPS is associated with an accumulation of chromatin activation modification at H3K4me3 (Bermick et al., 2017; Netea et al., 2020). In this study, we observed a significant induction of H3K4me3 expression in response to BCG and LPS treatment (*p* < 0.05) (Figure 5A). Notably, UEVp did not exert a significant influence on H3K4me3 expression. Conversely, UEVt and UEVc led to a reduction in activating H3K4me3 expression (Figure 5A). This suggests that UEVt exhibited a greater capacity than UEVp in suppressing this activating chromatin modification at H3K4me3. Alongside the increase in activating H3K4me3 expression, we also observed heightened levels of proinflammatory cytokines such as IL-6 (Figure 5B) and IL-8 (Figure 5C) in response to the trained immunity induced by BCG and LPS. Interestingly, UEVp, which did not suppress H3K4me3 expression (Figure 5A), had a significantly higher production of IL-8 (*p* < 0.001) (Figure 5C) and TNFα (Figure 5D) than their counterparts induced by UEVt.

## 4 Discussion

This study is the first to show that immune mediators of first-trimester maternal UEVs can predict which women are at risk of PB. Pregnant women have a higher number of UEVs than non-pregnant women. First-trimester UEVs from women with PB have significantly higher M1 proinflammatory mediators, including IL-6, IP-10, and MCP-1, than those with TB. The UEVs from women with TB better suppress the *ex vivo* BCG-trained M1 proinflammatory response than the UEVs from women with PB. These findings suggest that early altered M1 polarization in maternal UEVs may be involved in the programming of subsequent PB, and early modulation of trained M1 immunity by UEVs leading to better Treg differentiation may prevent subsequent PB.

Most previous studies on PB biomarkers have been carried out in maternal blood at the time close to or upon the onset of labor (Huang et al., 2004; Yang et al., 2004; Negi et al., 2012). There are only few studies on PB biomarkers in the early first trimester. EVs derived from the mother, placenta, and fetus are rich in blood during pregnancy, and the communications between the exosomes from the fetus and mother are implicated in normal and abnormal pregnancies (Holder et al., 2016; Yang et al., 2019; Czernek and Düchler, 2020). The particle number of plasma exosomes derived from pregnant women with gestational diabetes was higher than in normal pregnancy, and the size was also larger than that from normal pregnancy (Salomon et al., 2016). The mechanism for the higher and larger plasma exosomes derived from preeclampsia remains unknown. Some studies also reported that plasma exosomes derived from pregnancy with and without PB expressed different proteomic profiles (McElrath et al., 2019; McElrath et al., 2020). There are limited studies carried out with biomarkers of UEVs as a non-invasive liquid biopsy for pregnancies with and without PB. First-trimester urine metabolomics and proteomics for the prediction of preeclampsia have been reported in some studies (Austdal et al., 2015; Guo et al., 2019), showing lower sensitivity and specificity for the prediction of preeclampsia using metabolomics profiles or proteomic profiles. To the best of our knowledge, we are the first to demonstrate that altered proinflammatory mediators of UEVs in the first trimester could predict the subsequent occurrence of PB, and UEVs derived from women with PB and TB revealed different capabilities to suppress the BCG-trained M1 proinflammatory reaction. We also found that pregnant women had a significantly higher number of UEVs than those without pregnancy. Whether the higher number of UEVs is involved in successful pregnancy remains to be determined.

Both innate and adaptive immunities are involved in normal and abnormal pregnancies (Pavlov et al., 2008; Robertson et al., 2010; Lin et al., 2014; Xu et al., 2016; Romero et al., 2018). The pattern recognition receptor (PRR), such as Toll-like receptor (TLR), recognizing pathogen-associated molecular patterns (PAMPs) derived from microorganisms or damage-associated molecular patterns (DAMPs) from damaged and dead cells, has been linked to the inflammatory pathway causing PB, and the antagonist of TLR4 could prevent PB in animal studies (Chin et al., 2016; Romero et al., 2018). Recently, the “trained immunity” of innate immune cells at the fetal–maternal interface such as uterine mucosa possessed the ability to “remember” for subsequent successful pregnancies (Feyaerts et al., 2021). The trained immunity refers to the enhanced memory responsiveness of innate immune cells such as macrophages upon re-exposure to the same or different stimuli (Fanucchi and Mhlanga, 2019; Netea et al., 2020). For instance, the BCG-trained immunity of M1 proinflammation could restrict fetal growth in animal models (Dang et al., 2022), and decorin-mediated macrophage differentiation has been associated with pregnancy loss (Wang et al., 2022). Dysregulation of Th1-Th17/Treg has been linked with repeated pregnancy loss (Wang et al., 2020). Arenas-Hernandez et al. (2019) demonstrated that T-cell activation by anti-CD3 induced PB via the overexpression of IL-6 and IFNγ. In animal studies, IL-22 and IL-6 have been identified as factors related to PB (Gilman-Sachs et al., 2018), and abnormal dendritic cell population and activity have been implicated in recurrent abortion, which might be rescued by the immune modulation of baicalin (Lai et al., 2022). In a birth cohort study, IL-6 was a risk factor of PB in underweight pregnant women, while IL-2 was a protective factor of PB in overweight pregnant women (Curry et al., 2009). To our knowledge, this study is the first in the literature to delineate the biomarkers of first-trimester maternal UEVs, showing altered M1 differentiation with higher levels of MCP-1, IL-6, and TNFα, which were associated with the subsequent occurrence of PB. The fact that first-trimester UEVs derived from women with PB revealed significantly higher M1 and Th1 mediators than those from women with TB, and UEVs derived from TB could reverse the trained M1 polarization associated with a higher Treg differentiation, suggests that predominant M1 immunity reflected in the first-trimester UEV environment might progress to PB, and those with diminished M1 polarization with higher Treg differentiation might progress to normal TB.

This study has both advantages and limitations. On the positive side, this study employs a longitudinal cohort approach and utilizes a non-invasive liquid biopsy of first-trimester UEVs to forecast the progression of PB. However, there are limitations that need improvement and refinement. 1) This study lacks the simultaneous collection of maternal and umbilical cord blood for comparison in trained immunity. 2) The sample size is relatively small for the comprehensive analysis of PB predictors from diverse subtypes. 3) This study does not examine additional parturition factors in UEVs, such as placental growth factors like EG-VEGF, known to play a role in parturition (Dunand et al., 2014) and predicting PB (Raia-Barjat et al., 2023), or microRNA profiles that potentially differentiate PB prediction from TB (Winger et al., 2020). Further studies with larger cohorts are crucial to validate these findings and delve deeper into the potential of UEVs for influencing macrophage differentiation for the early prediction and prevention of PB.

**FIGURE 6:**
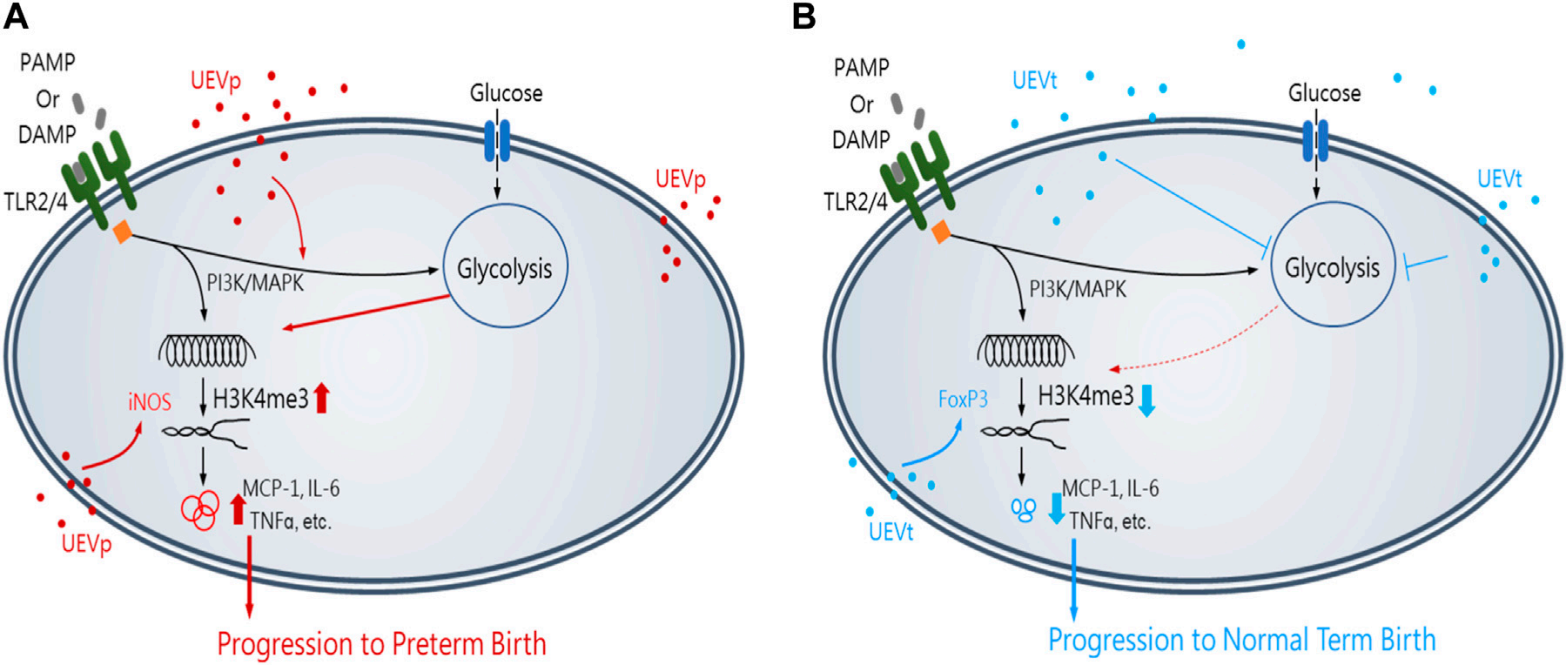
Insights of the first-trimester UEVs involved in immune programming of PB or TB. A pregnancy-related PAMP or DAMP, which mediates an altered immunity of M1 differentiation with iNOS induction and the activation of PI3K and MAPK for augmented glycolysis, resulting in the accumulation of activating chromatin modification of H3K4me3 and significantly elevated production of M1 proinflammatory mediators, such as MCP-1, IL-6, and TNFα, for progression to PB, in which UEVs from women with PB (nanovesicles in red) (UEVp) contained altered immune mediators to augment M1 (iNOS) expression **(A)**. In contrast, the UEVs from women with TB (nanovesicles in blue) (UEVt) contained few M1 proinflammatory mediators and demonstrated a better capacity to enhance regulatory FoxP3 expression and suppress the trained M1 proinflammatory response with glycolysis and lessen the accumulation of H3K4me3 expression for progression to TB **(B)**.

In summary, this study unveils groundbreaking insights into immune programming within first-trimester maternal UEVs as predictive markers for identifying women at risk of PB. Notably, pregnant women exhibited a higher abundance of UEVs than their non-pregnant counterparts. UEVs sourced from women with PB displayed significant elevation in M1 polarization and Th17 differentiation, marked by the heightened induction of inflammatory mediators and the expression of iNOS and RORrT, in contrast to their counterparts in UEVs from those with TB. The results of this study lead us to propose a conceptual framework illustrating the involvement of first-trimester UEVs in immune programming for PB or TB, as shown in Figure 6. This immune programming may be initiated by a pregnancy-related PAMP, such as LPS, or DAMP, such as cell-free DNA, which mediates altered signal transduction via Toll-like receptors (TLR2 or TLR4), leading to M1 polarization with heightened iNOS expression and Th17 differentiation, associated with augmented glycolysis of glucose consumption, accumulation of chromatin activation modification at H3K4me3, and elevated production of proinflammatory cytokines for progression to PB (Figure 6A). Conversely, UEVs from women with TB demonstrate a superior capacity to suppress trained M1 proinflammatory responses, enhance FoxP3 expression indicative of Treg differentiation, and reduce glycolysis, resulting in lower H3K4me3 accumulation and diminished induction of proinflammatory cytokines, as shown in Figure 6B.

This study suggests that PB may be predicted by elevated M1 and Th17 immune mediators in first-trimester UEVs. Early intervention to modulate M1 and Th17 immunity in UEVs for improved immune regulation could potentially hold promise in preventing subsequent preterm births.

## Data Availability

The original contributions presented in the study are included in the article/Supplementary Material; further inquiries can be directed to the corresponding author.

## Abbreviations

UEV, urinary extracellular vesicle; PB, preterm birth; TB, term birth; M1, type 1 macrophage; M2, type 2 macrophage; Th1, T helper cell type 1; Th2, T helper cell type 2; and Treg, T regulatory cell.
