# Supplementary material for: First-trimester urinary extracellular vesicles as predictors of preterm birth: an insight into immune programming
Source: Front Cell Dev Biol. 2024 Jan 31;11:1330049. doi: 10.3389/fcell.2023.1330049 (PMC10864598; doi:10.3389/fcell.2023.1330049)

Supplementary file

1. Supplementary data of original western blots for Figure1C

CD63 (with variant glycosylation)

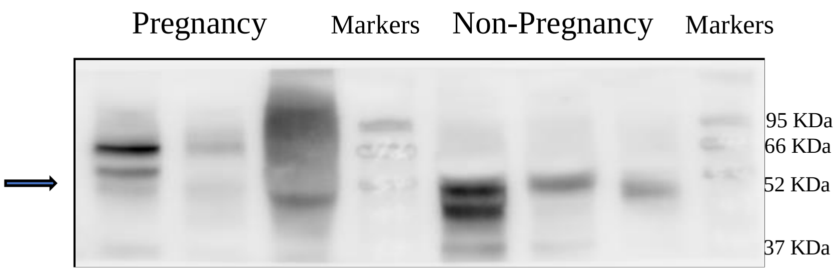

CD9

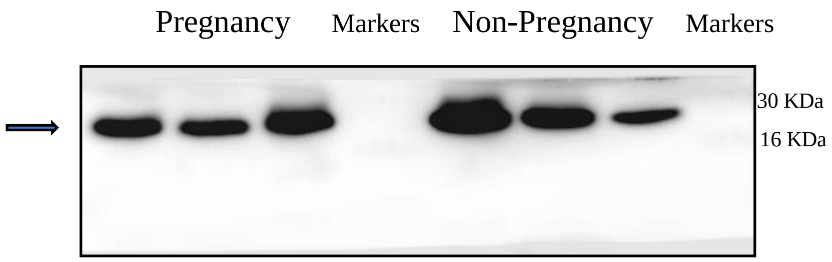

CD81

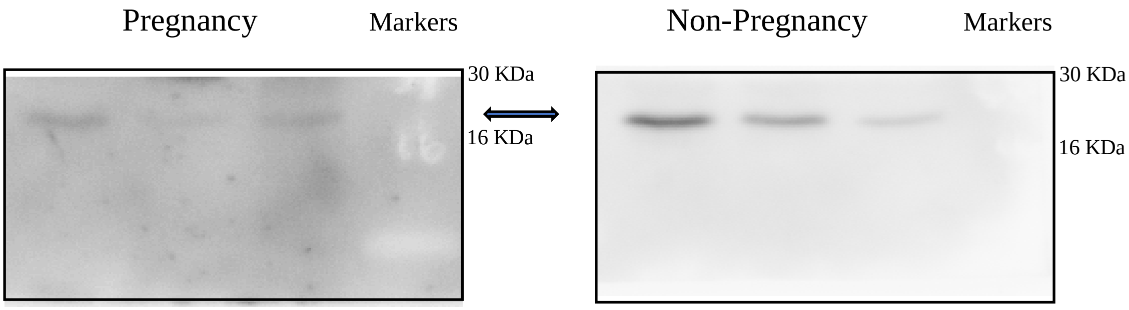

Syntenin

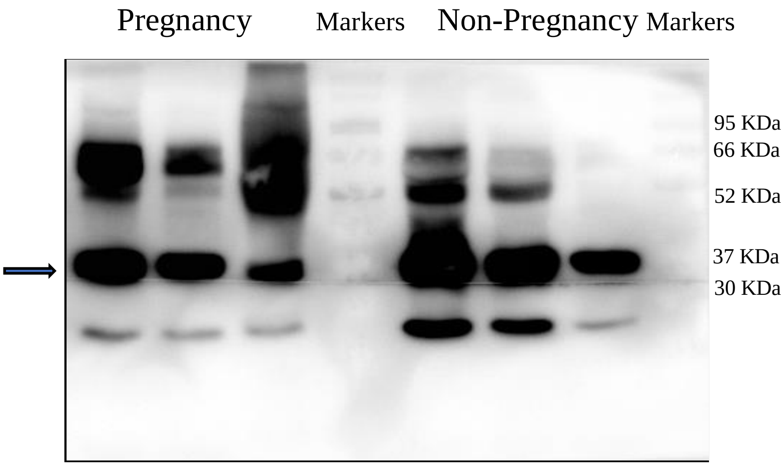

2. Supplementary data of original western blots for Figure 5A (4 Exp.)

Exp. 1

GAPDH

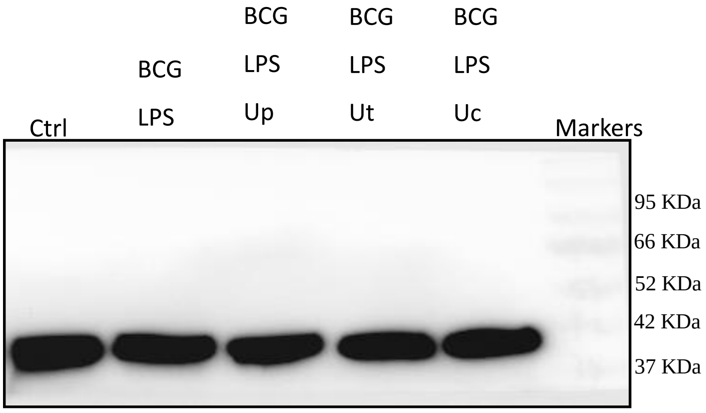

H3K4me3

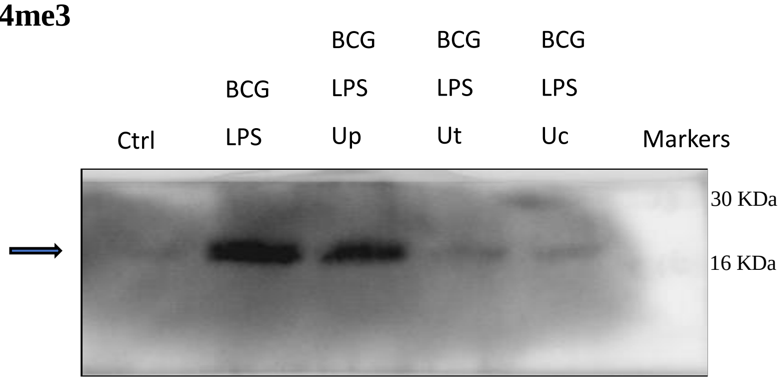

Exp. 2

GAPDH

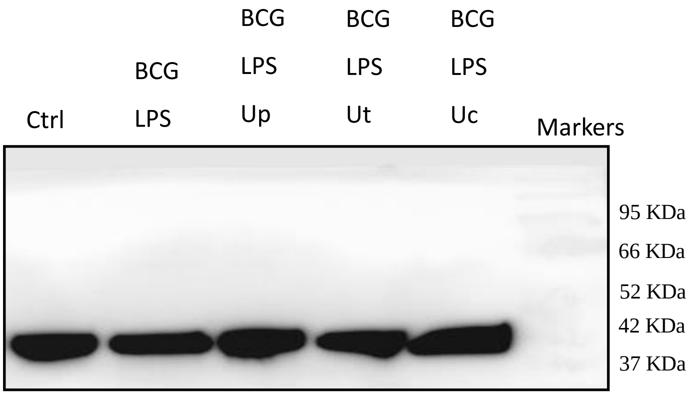

H3K4me3

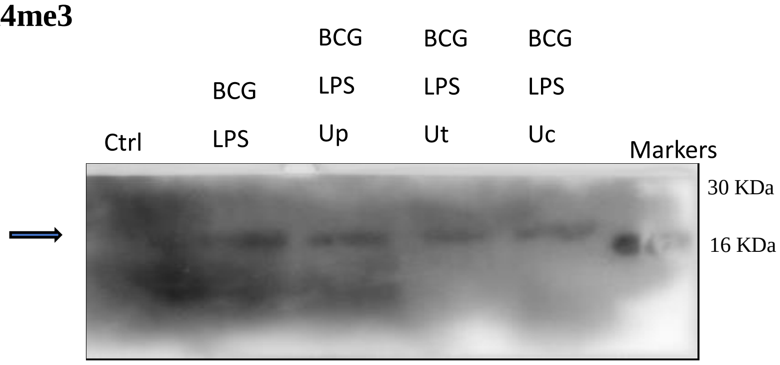

Exp. 3

GAPDH

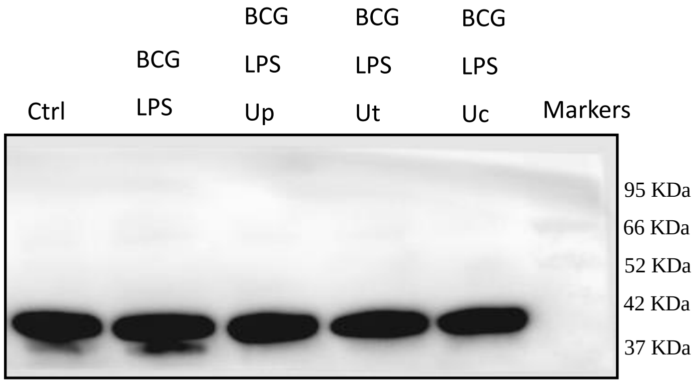

H3K4me3

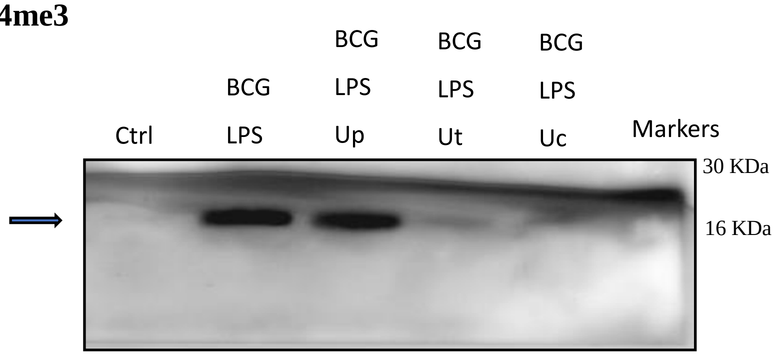

Exp. 4

GAPDH

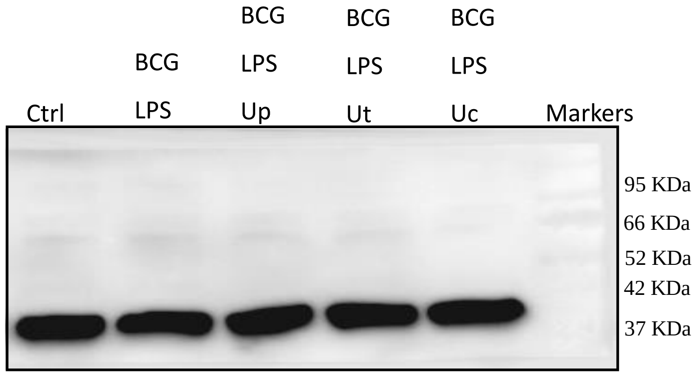

H3K4me3

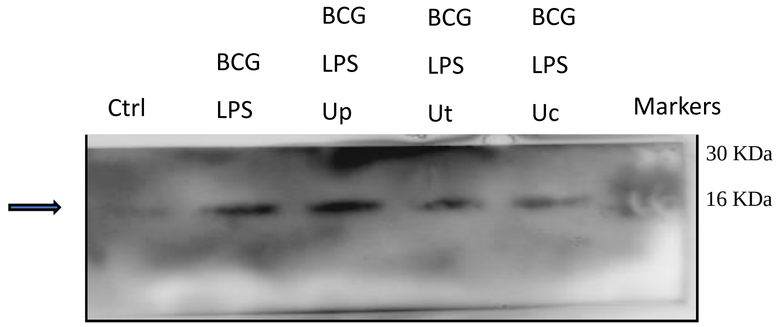

Supplement: Supplementary file 1 [file DataSheet1.PDF]
